# 

**Published:** 1887-03

**Authors:** 


### CONTRIBUTORS.

| | |
|---|---|
| J. J. Abrams, Esq | Savannah, Ga. |
| James B. Baird, M. D | Atlanta, Ga. |
| R. B. Barron, M. D | Clinton, Ga. |
| Clark Bell, Esq | New York. |
| J. Emmett Blackshear, M. D | Macon, Ga. |
| G. F. Blandford, M. D | London, Eng. |
| Nathan Bozeman, M. D | New York. |
| B. H. Brodnax, M. D | Brodnax, La. |
| W. L. Bullard, M. D | Columbus. Ga. |
| J. J. Buster, M. D | Mt. Willing, S. C. |
| A. W. Calhoun, M. D | Atlanta, Ga. |
| W. Cheatham, M. D | Louisville, Ky. |
| Hunter P. Cooper, M. D | Atlanta, Ga. |
| R. O. Cotter, M. D | Macon, Ga. |
| R. P. Cox, Esq | Rome, Ga. |
| J. M. Crawford, M. D | Atlanta, Ga. |
| Eugene Foster, M. D | Augusta, Ga. |
| J. W. Flanders, M. D | Wrightsville, Ga. |
| John D. S. Davis, M. D | Birmingham, Ala. |
| Nathan S. Davis, M. D | Chicago, Ill. |
| A. Freeman, M. D | New York. |
| R. E. Fuller, M. D | Macon, Ga. |
| J. Mc. F. Gaston, M. D | Atlanta, Ga. |
| James A. Gray, M. D | Atlanta, Ga. |
| J. Edward Green, M. D | Macon, Ga. |
| J. W. Griggs, M. D | West Point, Ga. |
| R. R. Harden, M. D | Harmony Grove, Ga. |
| Virgil O. Hardon, M. D | Atlanta, Ga. |
| T. J. Hargan, M. D | Asheville, N. C. |
| T. M. Holmes, M. D | Rome, Ga. |
| Harry Huzza, M. D | Rome, Ga. |
| W. J. Jolly, M. D | Waldo, Fla. |
| John L. Jones, M. D | New Orleans, La. |
| L. H. Jones, M. D | Atlanta, Ga. |
| W. S. Kendrick, M. D | Atlanta, Ga. |
| E. W. Lane, M. D | Scarboro, Ga. |
| C. B. Leitner, M. D | Flora, Ala. |
| Joseph M. Mathews, M. D | Louisville, Ky. |
| H. V. M. Miller, M. D., LL. D | Atlanta, Ga. |
| H. C. Morrison, D. D | Atlanta, Ga. |
| H. N. Moyer, M. D | Chicago, Ill. |
| W. B. Parks, M. D | Atlanta, Ga. |
| S. Latimer Phillips, M. D | Savannah, Ga. |
| F. M. Ridley, M. D | LaGrange, Ga. |
| J. M. Sanders, M. D | Magnolia, S. C. |
| W. J. Scott, D. D | Atlanta, Ga. |
| H. H. Smith, M. D | Philadelphia, Pa. |
| Albert B. Strong, M. D | Chicago, Ill. |
| V. H. Taliaferro, M. D | Atlanta, Ga. |
| Lawson Tait, F. R. C S | Birmingham, Eng. |
| J. S. Todd, M. D | Atlanta, Ga. |
| Robert Tilley, M. D | Chicago, Ill. |
| W. F. Westmoreland, Jr., M. D | Atlanta, Ga. |
| Henry Wile, M. D | Atlanta, Ga. |
| H. J. Williams, M. D | Macon, Ga. |

SOCIETIES.

Atlanta Society of Medicine.
Chicago Medical Society.
Medical Association of Georgia.
Baltimore Academy of Medicine.
Clinical Society of Maryland.
Ninth International Medical Congress.
Philadelphia County Medical Society.

### INDEX.

A

Abortion, criminal 314, mongers, the punishment of 242.
Abscess of the brain, A case in practice supposed to be an 286, with cases 604.
Abscess, Retropharyngeal, with report of two cases of 405.
Aid to the injured 423.
Alcoholism, chronic 684.
Alcoholic liquors in the practice of medicine 257, 320, 3[illegible]5, 389.
Alcohol poisoning 282.
Ambulance, Service of New York 224.
A monstrosity 203.
Anatomical Board, The State 433, 687.
Annual address of the President of the Jefferson Co Medical Society 715.
Annual opening 557.
Antipyrin and Antifebrin in the treatment of nervous diseases 626.
Atheroma of the left coronary artery resulting in aneurism of the apex of the left ventricle 526.
Atlanta, Health of 50, the cost of caring for the sick—poor of 316.
Atlanta Medical College 498, an address to the graduating class of 65.
Atlanta Medical and Surgical Journal 495.
A reply to Dr. Battey and a disclaimer 31.

B

Bacteria in water and ice 181.
Big fees in New York 225.
Blood vessels, calcification of, followinginjury 88.
Book Reviews: Anæmia 769—A Manual of treatment by Massage 371, Anatomy—Descriptive and Topographical 490, A reference hand-book of the Medical Sciences 107, 630, Athothis—A satire on modern medicine 426, A text-book of Medicine (Bartholow) 239, of Medicine (Strumpell) 236, of Pathological Anatomy 365, of Surgery 103, A treatise on compound Ophthalmic Lenses 112, A treatise on Diphtheria 631, Bryant's operative Surgery 48, Cyclopaedia of Obstetrics and Gynecology 234, 311, Diagnosis and treatment of Hemorrhoids 554, Diseases of the blood and nutrition 49, Diseases of the Lungs and pleuræ, including Consumption 110, Diseases of the Hair and Scalp 770, Drug Eruptions 370, Druitt's Surgeons' Vade-Mecum 628, Earth as a topical application in Surgery 369, Gray's Anatomy 554, History of the Confederate States Navy 490, Lessons in Gynecology 552, Maternity infancy and childhood 426, Massage 770, Medical Electricity 371, Medical and Surgical memoirs 104, Nervous Diseases and their Diagnosis 110, Outlines for the management of diet 425, Pathology and treatment of Gonorrhœa and Spermatorrhœa 487, Physicians' visiting list 630, Practical lessons in nursing 367, Practitioners' hand-book of treatment 368, Smith's operative surgery 48, Students' guide to diseases of the Eye 630, Transactions of the Association of American Physicians 368, 769, Vest Pocket Anatomist 490, Wear and Tear 367, What to do in cases of poisoning 425.
Brain, Abscess of the—A case in practice supposed to be an 286, with cases 604, a large tumor of 181
Burial Reform Association 765.

C

Caries, spinal, the early diagnosis of 517.
Cataract extractions and Iridectomies after treatment of 645.
Cellulities, Pelvic, some observations upon 453.
Chalazion 473.
Children, brain-forcing in the education of 44, the poor and how they are cared for 306.
Chicago, a letter from 420.
Cholera, Asiatic in New York 545, infantum 271.
Chronic alcoholism 684.
Civil Service examinations for medical positions 357.
Colic, infantile, quinine in 193, hepatic, dioscorine in 195.
Color blindness 430.
Conjunctivities, granular, with and without pannus 142.
Coronary artery, atheroma of the, resulting in aneurism of the left ventricle 526.
Country Doctor, a letter from 309.
Cremation—progress of 765.
Criminal abortion 314.

D

Delerium, a peculiar case of 358.
Dioscorine in hepatic colic 195.
Diphtheria, its cause and treatment 647.
Doctors can practice dentistry 417.
Doctors jailed, two 55
Doctor's shop of the olden time 561.
Does it favor Sam. 248.
Dysentery, Rockbridge alum water in 198.

E

Eczema, treatment of 1.
Elbow, treatment of old dislocations of 179.
Empyema, Subperiosteal resection of a rib for 729.
Enucleation, indications for and against 588.
Epilepsy 306.
Episcleritiis 337.
Ergot in internal hemorrhoids 196, 311.
Expert testimony 178.
Eye, foreign bodies in 472, transplanting a rabbit's to a human subject 98.

F

Fever, hay 305. Remittent, its prompt arrest and effective treatment 83, 137.
Fistula in Ano, the relation of phthisis pulmonalis to 709.

Florida, a trip through 182.
Fracture, report of two cases of 531.
Fraud exposed 559.

G

Gaston, Dr., explains 307.
Gleditschine, investigation of 635.
Gonorrhœa, speedily relieved 654.

H

Hay fever 305.
Hemorrhoids (internal), fluid extract of ergot in 196.
Herpes facialis, progentialis 8, zoster 129.
Hydrastis Canadensis in uterine troubles 738.

I

Insane, investigation of abuse of 624.
Insanity, the treatment of recent cases of in asylums and private houses 597.
Intra-uterine Therapeutics 427.
Intemperance to Insanity, The relation of 655.
Iodine—colorless, Tincture of 321.
Iridectomies and cataract extractions. After treatment of 645.
Iritis sympathetic 15.
Items: 14, 52, 122, 193, 209, 222, 243, 285, 289, 303, 320, 340, 376, 431, 498, 562, 635, 687.

K

Keratosis obturans 592.
Knowing too much 633.

L

Lachrymal duct, The superiority of large probes in the treatment of stricture of 471.
Larynx, intubation of 304.
Laws regulating the Licensing of Physicians and the practice of Medicine 771.
Lichen ruber 136.
Life force, A new theory of 502.
Lying-in charity, The Philadelphia 360.

M

Malarial manifestations due to traumatism, A case of 339.
Mammitis, polk root in 197, 309.
Medico-Legal subjects 74.
Medical Association of Alabama 125.
Medical Association of Georgia 114, 147, 186, 190.
Medical charity, abuse of 96.
Medical College, Atlanta commencment of 125.
Medical College, Southern commencement of 123.
Medical Congress, ninth international 102, 374.
Medical reforms 419.
Malaria 132.
Milk Sickness the cause of 766.
Mineral waters 240.
Mitral Regurgitation 747.
Mistletoe 197, 309.
Morgue, a new plan for 485.
Myxomata of the orbit 595.

N

Neurasthenia 9.
Neuritis multiple 341.
Its relation to certain peripheral neuroses 46.
Nose, diseases of the 581.

O

Obituary—Dr. James A. Gray 492, 576, Dr. V. H. Taliaferro 497, Dr. John G. Westmoreland 119, Dr. Henry Wile 191.
Obstetrics, a unique result in 627.
Opium smoking 680.
Orbit, myxomata of the 595.
Our advertisers 63, 128, 196, 256, 323, 387, 452.

P.

Parotiditis, a case of metastatic, terminating in suppuration, recovery 205.
Pelvic cellulitis, some observations upon 453.
Pemphigus Foliaceus 134, Vulgaris 133.
Perineum, laceration of the 403, 550.
Peritonitis Septic in the new-born 764.
Personal: Drs. W. S. Armstrong 376, A. B. Ashworth 687, Robert Battey 499, 687, J. Emmett Blackshear 512, L. E. Borcheim 511, D. G. Brinton 244, C. A. Brooks 376, A. W. Calhoun 432, Hunter P. Cooper 688, 772, W S Elkin 320, J G. Faircloth 568, Eugene Foster 512, J. McF. Gaston 193, D. W Hammond 376, N. O. Harris 512, T. S. Hopkins 243, 432, Harry Huzza 193, John Thad. Johnson 821, 772, Woolsey Johnson 358, W. S. Kendrick 321, 512, 695, J. F Lancaster 431, Joseph P. Logan 321, F. W. McRae 193, H. V. M. Miller 431, G. H. Noble 193, A. G. North 122, J. B. Pendergrass 54, N. A. Randolph 432, J. A. Reagan 564, E. H. Richardson 54, T. B. Rider 687, V, H. Taliaferro 376, W. A. Strother 54, J. S. Todd 320, W. F. Westmoreland, Jr. 122, 320, A. G. Whitehead 244, 321, Henry Wile 123, W. C. Wile 55, Miss J. W. Ainger 377, George A. Shepherd 125.
Physician and patient, confidential relations between 113.
Physicians in New York, young 96.
Phthisis catarrhal 60.
Phthisis Pulmonalis, the relation of Fistula in Ano to 709.
Pneumonia, treatment of 652.
Poisoning by illuminating gas treated by rectal injections of oxygen 542.
Pompholyx 133.
Post graduate study 556.
Pregnancy, Mechanical treatment of Vomiting in 733.

Q

Quinine in infantile colic 194.

R

Remittent fever, its prompt arrest and effective treatment 83, 137.
Rhinitis atrophic, the galvanic current in the treatment of 305.

S

Sarcomagiant cell, result of the removal of 124.
Sciatica, the relation of to organic stricture of the urethra 736.
Shall medicines be administered before or after meals 199.
Should preachers pay 41, 230.
Sloane Maternity Hospital 763.
Sims monument, the 98.
Skin grafting, medico-legal aspects of 52.
Society Reports: Atlanta Society of Medicine 19; Baltimore Academy of Medicine 672, Chicago Medical Society 23, 210, 290,

353, 407, 534, 753, Clinical Society of Maryland 90, 347, 667, Medical Association of Georgia 147, Ninth International Medical Congress 474, Philadelphia County Medical Society 613.
Spinal caries, the early diagnosis of with remarks on treatment 517.
Subperiosteal resection of a rib for empyema in children 729.
Symphysis pubis, seperation of during parturition 184.
Syphilis, treatment of 126, 682.
Systole, heart shortened in 45.

T

Taliaferro and Bozeman controversy 35, 100.
Thou shalt not steal 116.
The Candler bill 373.
The relation of Fistula in ano and Phthisis Pulmonalis 725.
The relation of sciatica to organic stricture to of the urethra 736
Tumor, a large brain 181.
Trachelorraphy—Superinvolution of the uterus following—663.
Traumatism, a case of malarial manifestations due to 339.
Twins, an interesting case 545.
Typhoid fever, increase of in New York 419.

U

Uterine therapentics, Intra 427.
Uterus, superinvolution of the following trachelorraphy 663.

V

Vomiting in pregnancy—mechanical treatment of 733.

W

What shall be the color of our summer clothing 484.
Workingmen, the food of 484..

Y

Yellow fever, appropriation for the investigation of 117, inoculation against 226, status of 467.

### OUR ADVERTISERS.

SURGICAL INSTRUMENTS.—Mr. Isaac Phillips, at 14 Marietta street, Atlanta, has the largest assortment of surgical instruments and appliances to be found anywhere in the South, and he says he is offering them at prices far below anything ever offered before. See his advertisement and call on him or write for prices.

CHAS. O. TYNER, ATLANTA, GA.—This gentleman has in connection with his drug business a full line of surgical instruments, to which he invites the attention of the profession by an attractive advertisement which appears elsewhere. He says he can and will sell goods as low as can be sold anywhere. Call on him and get his prices.

VIN MARIANI COCA.—Professor Marius Odin, M. D., contributes to the *Gazette de Therap.* an interesting paper on the value of this wine in cases broken down from long-continued disease, or for persons worn down by over-work. We have witnessed some excellent results from the use of the wine in such cases, and can unhesitatingly recommend it.

A SUCCESSFUL FOOD FOR INFANTS.—Douglass H. Stewart, M. D., 332 W. 47th street, New York, reports as follows: "I have made a test in above fifty cases of the lactated food, kindly sent to the Northwestern Dispensary for me, and can only add that in every instance there was an improvement more or less marked. I have had such poor success with '——,' '——' and kindred foods that I employed your preparation in rather a faint-hearted way at first, but after one or two trials was convinced that lactated food is all you claim for it."

T. J. REID, Chicago, Ill., says: Syrupus Roborans is an excellent tonic and assimilates nutrition very rapidly. Being a perfect solution of the hypophosphites and strychnia, must necessarily commend it to the medical profession as equal, if not superior, to any compound of the kind in the drug market. I have tried it in some cases of phthisis pulmonalis and chronic anæmia with the most gratifying results. As a reconstructing agent it must become a prominent stable preparation and a boon to all who are emaciated and enfeebled from the effect of chronic diseases of the blood and nervous system.

CARLSBAD WATERS IN THE TREATMENT OF GOUT AND BILIOUSNESS.—Mr. B. J., æt. 69, weight 190 pounds, height 5 feet 7½ inches, had an attack of renal colic about twenty-two years ago. Oxalate crystals appeared in the urine. For a time his life was despaired of. Fifteen years ago he began to grow stouter, and had periods of depression. He consulted a physician at that time, who advised horseback exercise; otherwise, serious results might follow. This instruction was not carried out. Eleven years ago, while being treated for a Colles fracture, he had an attack of inflammatory rheumatism confined to the right shoulder joint. Shortly after this he had another attack of renal colic, not as severe as the first one. Since 1860 he has had occasional attacks of muscular rheumatism, at times quite severe. In the early part of last year he began to complain of pain in the thumbs, which were swollen. He had pain in the toes, but not so marked. The pain in the thumbs kept him awake at night; he takes very little exercise. Diagnosis: Gout. He was put on a course of iodide of potassium and the wine of colchicum seed without beneficial results, although pursued for a long time. The salicylates were then tried with the same result. Both the above methods were pursued in conjunction with a regulated diet. He was then placed on Carlsbad waters, the Sprudel being used. He drank three or four wineglassfuls a day, the water always being heated first. Beneficial results were noticed before beginning the second bottle, as the patient was able to sleep at night. Improvement continued, and after six bottles had been used, the pain was gone and the swelling disappeared. At the present writing, six weeks after this treatment was begun, the patient is perfectly well, having used but ten bottles of the water. The only restriction placed upon Mr. B. J. while drinking the Carlsbad waters was that he was not to drink any malt and very little of spirituous liquors. This he did not carry out, as he drank about a glass of beer a day.

Mrs. S. B., æt. 45, of full habit, has had frequent attacks of biliousness. Was called in to attend her early last fall. Symptoms as follows: Complexion muddy; tongue coated with yellowish fur; bitter taste in the mouth: appetite poor; bowels constipated; some dizziness. Her feet are somewhat swollen. She was given small doses of calomel, followed by a saline purge. Bitter tonics were then administered. Jalap, fifteen grains, and cream of tartar an ounce, were given about every four days to keep the bowels open and to diminish the swelling of the feet. The patient improved, and I discontinued my visits. About six weeks later I was sent for, when I found a recurrence of the above symptoms. The patient is very stout. On physical examination I found the liver considerably enlarged. Carlsbad Sprudel Water was ordered in wineglassful doses four times a day *to be taken cold.* The patient soon began to improve. After taking five bottles is well. The swelling of the feet has disappeared; the bowels are regular.

Mr. S. H., æt. 40, rather plethoric, exhibited symptoms similar to Mrs. B. Feet were not swollen, liver not enlarged. The same treatment was pursued with a regulated diet; was well after taking three bottles.

J. B. POTSDAMER, M. D.,

No. 1629 8th Street, Philadelphia.

